# ArrayPitope: Automated Analysis of Amino Acid Substitutions for Peptide Microarray-Based Antibody Epitope Mapping

**DOI:** 10.1371/journal.pone.0168453

**Published:** 2017-01-17

**Authors:** Christian Skjødt Hansen, Thomas Østerbye, Paolo Marcatili, Ole Lund, Søren Buus, Morten Nielsen

**Affiliations:** 1 Center for Biological Sequence Analysis, Department of Bio and Health Informatics, Technical University of Denmark, Kgs. Lyngby, Denmark; 2 Laboratory of Experimental Immunology, Faculty of Health Sciences, University of Copenhagen, Copenhagen, Denmark; 3 Instituto de Investigaciones Biotecnológicas, Universidad Nacional de San Martín, Buenos Aires, Argentina; Universita degli Studi di Catania, ITALY

## Abstract

Identification of epitopes targeted by antibodies (B cell epitopes) is of critical importance for the development of many diagnostic and therapeutic tools. For clinical usage, such epitopes must be extensively characterized in order to validate specificity and to document potential cross-reactivity.

B cell epitopes are typically classified as either linear epitopes, i.e. short consecutive segments from the protein sequence or conformational epitopes adapted through native protein folding. Recent advances in high-density peptide microarrays enable high-throughput, high-resolution identification and characterization of linear B cell epitopes. Using exhaustive amino acid substitution analysis of peptides originating from target antigens, these microarrays can be used to address the specificity of polyclonal antibodies raised against such antigens containing hundreds of epitopes. However, the interpretation of the data provided in such large-scale screenings is far from trivial and in most cases it requires advanced computational and statistical skills. Here, we present an online application for automated identification of linear B cell epitopes, allowing the non-expert user to analyse peptide microarray data. The application takes as input quantitative peptide data of fully or partially substituted overlapping peptides from a given antigen sequence and identifies epitope residues (residues that are significantly affected by substitutions) and visualize the selectivity towards each residue by sequence logo plots. Demonstrating utility, the application was used to identify and address the antibody specificity of 18 linear epitope regions in Human Serum Albumin (HSA), using peptide microarray data consisting of fully substituted peptides spanning the entire sequence of HSA and incubated with polyclonal rabbit anti-HSA (and mouse anti-rabbit-Cy3). The application is made available at: www.cbs.dtu.dk/services/ArrayPitope.

## Introduction

The highly diverse repertoire of antibodies constitutes a very important component of the immune-mediated protection against pathogens. The exquisite target specificity and high affinity of binding make antibodies attractive tools in scientific, diagnostic and therapeutic applications. Characterization of the specificity of antibodies towards their binding site (epitope) is important for their selection towards intended targets and preventing unintended cross-reactivity [1].

Protein epitopes are usually classified as linear or conformational, depending on whether the amino acids comprised are brought together by proximity in the peptide chain or by protein folding, respectively [2]. The majority of epitopes are thought to be conformational, but the distinction is not clear-cut since conformational epitopes often contain small segments of continuous residues able to bind the antibody on their own [3,4]. Since conformational epitopes rarely maintain readily detectable binding activity outside the context of the native protein structure, characterization of the conformational epitopes can be an extremely difficult task. On the other hand, linear epitopes (and linear segments of conformational epitopes) can be characterized by studying antibody binding to short peptide fragments of the protein.

Characterization of the specificity of polyclonal antibodies toward any potential linear epitope within an antigen is challenging. Many different methods including solid phase peptide libraries and phage displayed peptide libraries [5,6] have been used to screen for linear epitopes. Although peptide display systems offer high-throughput identification of linear mimotopes [7] they have biases with regard to certain sequence populations and selection steps [6,8,9]. Using synthetic peptides to map target antigens, the mapping resolution depends on the length and overlap of the analysed peptides as well as subsequent truncations or substitutions, used to fine-map the location of the epitope and the contribution of the individual amino acids to the antibody binding [10]. Most studies based on synthetic peptides involve mapping of native-sequence proteins using overlapping peptides of length 15–30 amino acids [11,12] and some use alanine scans, in which alanine substitutions are introduced in the synthetic peptides to improve mapping resolution [13].

Recent advances in high-density peptide microarrays have enabled parallel synthesis of hundreds of thousands of peptides [10,14]. Two studies have used peptide microarrays to conduct full-resolution epitope mapping using exhaustive single-amino acid substitution analysis [10,15]. These studies use statistical methods to pinpoint which residues of a given peptide epitope are involved in antibody interaction, by identifying significant changes in signal intensity upon substitutions relative to the native sequences. By inspecting the individual amino acid substitutions at the different positions within a given peptide the antibody specificity can be characterized. However, microarray-driven amino acid substitution analysis can be quite cumbersome, e.g. when mapping epitopes in hundreds of proteins this way.

Here, we present *ArrayPitope*, an automated analysis tool for characterizing and visualizing the specificity of the epitopes identified through large-scale single-amino acid substitution analysis of peptides. The tool identifies the contribution of each amino acid residue of the target protein for recognition of the corresponding antibodies and subsequently incorporates binding signals from overlapping peptides into one statistical analysis to precisely map the selectivity of each residue of the target protein involved in the recognition. As an illustration of the utility of this tool, we apply the method on a full-scale substitution analysis of the 69 kDa human serum albumin to address the specificities of polyclonal antibodies raised against the protein. An online implementation of the tool has been made freely available at www.cbs.dtu.dk/services/ArrayPitope.

## Methods

The method takes quantitative peptide data and a set of protein sequences as input. All peptides are mapped back to the protein sequence including all single-amino acid derivatives of these peptides. The intensity values of peptides subjected to substitutions are rescaled relative to the intensity of corresponding native peptide (median is used if multiple copies of native peptide exist). As such, substitutions giving rise to a lower intensity relative to the native peptide result in substitution values less than 1, and substitutions with no effect on the intensity takes the substitution value of 1, whereas substitutions giving rise to a higher intensity relative to the native peptide (also known as heteroclitic responses) takes a substitution value more than 1. The signal variance is estimated from the pool of native peptides sampling N peptides using bootstrap method where N is the number of copies of the given native peptide. The algorithm next performs the statistical analyses in two steps: i) first, by calculating the statistical significance of the mean substitution effects of each position in individual native peptides to determine which peptide positions are part of the epitope, and next ii) the algorithm incorporates information from overlapping peptides containing a given position in the mapped protein and determines the position specific binding selectivity of the antibody. The two steps are outlined below.

### Epitope-calling in individual native peptides

For each native peptide sequence, the substitution values are used to generate a position-specific scoring matrix (PSSM), in which columns represents positions in the peptide and rows represents amino acid substitutions. To determine if peptide positions undergoing substitution lead to a disruption of antibody binding, the importance of each peptide position is inferred by a Dunnett’s test, i.e. comparing multiple sample means to a control population. Here the mean substitution value is compared to the theoretical value, *μ*_0_ = 1, of no selectivity. When *S*^*2*^ is the pooled variance of the PSSM, then ${SE}_{i}=\sqrt{S^{2}/n_{i}}$ is the pooled standard error of the i-th column of the PSSM, where *n*_*i*_ is the number of substitutions in column *i* of the PSSM. In a complete amino acid substitution analysis *n*_*i*_ (and thus *SE*_*i*_) is the same for all positions. The least-significant-difference (LSD) is computed as:

$${LSD}_{i}=t_{d}{SE}_{i}.$$

The critical value *t*_*d*_ is computed as the quantile for the one-tailed noncentral Dunnett’s test distribution corresponding to i) a given significance level, ii) the number of groups *k*, equal to the peptide length, minus 1 and iii) the number of degrees of freedoms equal to the number of substitution values minus the length of the peptide (∑*n*_*i*_ − *k*). Peptide positions, where the relative change in signal exceeds the LSD value, i.e *1-μ*_*i*_ *> LSD*_*i*_, are characterized as being part of the epitope.

### Determining the selectivity of positions in overlapping peptides

**Fig 1:**
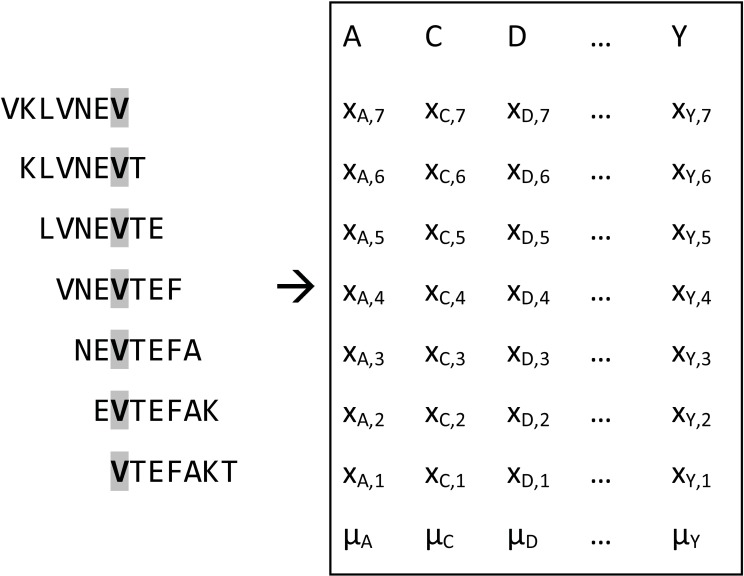
Example of substitution matrix. Figure showing overlapping peptides (left) with the valine (V highlighted in bold) being analyzed for antibody selectivity. The box contains the substitution values, *x*_*i*,*j*_, with columns representing the amino acid, *i*, which substitutes valine and rows representing the position, *j*, of the valine in the peptide (illustrated to the left) undergoing substitution. The mean substitution value, *μ*_*i*_, of each replacing amino acid is shown in the bottom row. The global mean, *μ*_*g*_, is calculated across all substitution values in the matrix.

For each residue in the protein being mapped in overlapping peptides, the algorithm seeks to determine which amino acid substitutions leads to a significant change in signal intensity relative to the native amino acid. Here, the substitution values are used to generate a substitution matrix expressing substitution values of *one* protein residue being represented in different positions in the overlapping peptides (see Fig 1 for a schematic illustration of the procedure). Here, only peptides are included containing protein positions previously identified to be involved in the epitope.

A Dunnett’s multiple comparison procedure is used to test which mean, *μ*_*i*_ (i.e. which replacing amino acid), that are significantly different from the value 1 of no selectivity. The LSD of each column of the substitution matrix is calculated as described above for each column of the PSSM. Protein positions will be reported and visualized, where the relative change in signal of one or more amino acid substitutions exceeds the LSD value.

**Fig 2:**
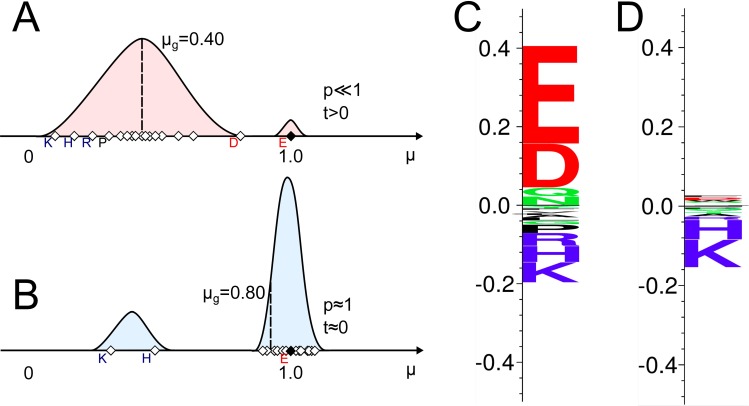
Schematic example of the generation of selectivity logo-plots. Figure showing example of selectivity of two epitope positions in overlapping peptides upon substitutions with all 19 amino acids. **A** and **B** shows illustrative density plots of substitution values taken from a substitution matrix, with the mean substitution value,.*μ*_*i*_, of each replacing amino acid shown in diamond shapes. Filled diamond is the native amino acid (E in both examples). **C** and **D** show the resulting logo-plot of the positions exemplified in A and B, respectively. The logo-plots are made using Seq2Logo [16].

If *SE*_*i*_ is the pooled standard error of the i-th column of the substitution matrix, then *t*_*i*_ = (*μ*_*i*_ − *μ*_*g*_)/*SE*_*i*_ is the t-statistic used to test the departure of the replacing amino acid *i* relative to the global mean, *μ*_*g*_, of the substitution matrix. Thus, mean substitution values above *μ*_*g*_ yield positive t-statistics (amino acids favouring interactions), while substitution values below *μ*_*g*_ yield negative t-statistics (amino acids disfavouring interactions). The p-value of the t-statistic is calculated from the cumulative distribution function for the noncentral Dunnett’s test distribution with degrees of freedoms equal i) to the number of replacing amino acid (up to 19) and ii) the total number of substitution values minus the number of replacing amino acids. To visualize the selectivity profile, each protein residue is presented in a logo-plot with the corresponding amino acid substitutions scored as

$$s_{i}=-sign(t_{i}){log}_{10}(p_{i}),$$

and subsequently rescaled so that ∑|*s*_*i*_| = (1 − *μ*_*g*_), consequently making the absolute sum of logo-heights reflect the mean change caused by substitutions of the native amino acid. To illustrate this, a sample data is shown in Fig 2, exemplifying two positions with high and low selectivity, respectively. In Fig 2A, only the native amino acid E (highlighted in solid fill at μ = 1) retains complete antibody binding (*μ*_*E*_ ≈ 1). The majority of the remaining amino acid substitutions lead to a decrease in signal and thus lower substitution value. The p-value associated with the native amino acid E is hence low (*p* ≪ 1), since the departure from the global mean *μ*_*g*_ is high (*t* > 0), leading to a high positive score, *s*_*E*_. The resulting logo-plot is shown in Fig 2C. The native amino acid employs the largest letter scale, but both the negatively charged amino acid D and the positively charged amino acids K, H and R employ larger letter scales due to their departure from *μ*_*g*_, in opposite directions. The absolute sum of the logo-plot column corresponds to the global effect of substitution (1 − *μ*_*g*_ = 0.60). Fig 2B exemplifies positions with only two amino acid substitutions affecting the signal. Here, the native amino acid, which also happens to be E (highlighted in solid fill at μ = 1) will employ a high p-value (*p* ≈ 1), since the substitution value is close to *μ*_*g*_, leading to a low score (*s*_*E*_ ≈ 0). The resulting logo-plot is shown in Fig 2D. The absolute sum of the logo-plot column is much smaller in this example (1 − *μ*_*g*_ = 0.20) and only substitutions to K and H are affecting the signal, as seen by the relatively large negative scales.

## Results

The ArrayPitope webserver was implemented to perform statistical analyses of the effects of single-amino acid substitution on a receptor-ligand interaction. Here, the webserver has been used to automatically map and characterize linear antibody epitopes in the human serum albumin (HSA) protein. Quantitative peptide microarray data was obtained from Hansen et al. [17] consisting of overlapping 15-mer peptides mapping the primary sequence of HSA (uniprot P02768) in 50 copies, including one copy of all possible single-amino acid substitutions. In total, the data consisted of 215,147 peptides. These peptides were measured for response to a commercially available polyclonal rabbit anti-HSA antibody using a secondary Cy3-conjugated goat anti-rabbit IgG, and fluorescence microscopy.

### Identifying peptide residues involved in antibody binding

**Fig 3:**
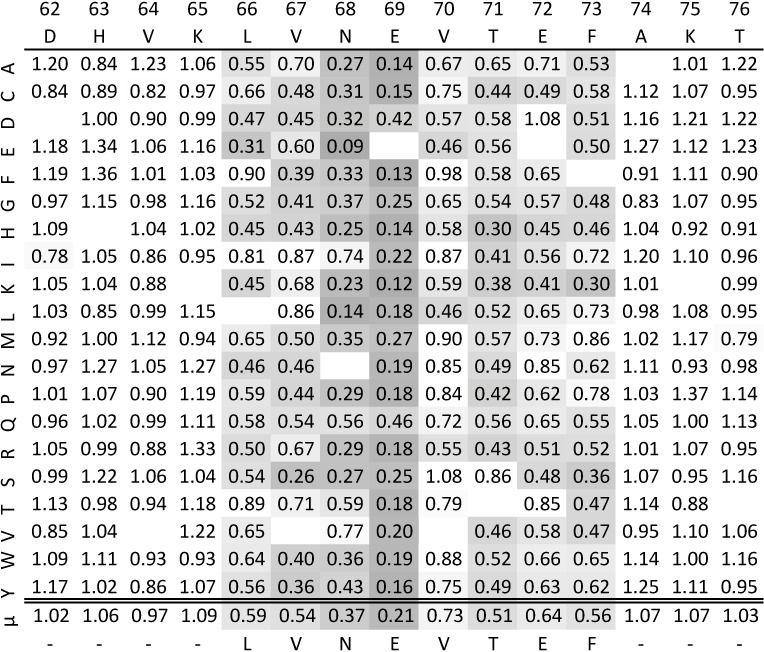
Example of a position specific scoring matrix. The table shows the individual substitution values for the peptide DHVKLVNEVTEFAKT through all 15 positions (columns) with all 20 amino acids (rows). The last row shows the mean substitution value of the positions. Blank cells are native amino acids. A Dunnett’s LSD of 0.1741 was calculated on the chosen p<0.0001 level and cells exceeding this difference are highlighted with a gray gradient (weak to strong effects). The identified epitope is LVNEVTEF.

To identify which peptides and peptide residues are involved in epitopes, a complete single-amino acid substitution analysis was performed by first constructing a position specific scoring matrix (PSSM) for each overlapping native peptide in the microarray data (see materials and methods for details). Fig 3 shows such PSSM for the peptide DHVKLVNEVTEFAKT, mapping position 62–76 of the primary sequence of HSA. The PSSM contains substitution values (relative to the native peptide) of all 19 replacing amino acids in all 15 positions of the peptide. For each PSSM, a Dunnett’s least-significant-difference was calculated for every position in the peptide, to determine if the mean substitution value is significantly different from the theoretical value 1 of no selectivity. For the purpose of demonstration, the significance level of 0.0001 was chosen for the following analyses. For the peptide shown in Fig 3, the positions 66–73 (spanning the octamer LVNEVTEF) were each identified to show a significant reduction (p<0.0001) in binding signal upon substitution.

The first part of the output of the algorithm is a table of all the overlapping peptides mapping the target protein(s) with their identified epitope highlighted. A limited part of the output table for HSA is shown in Table 1, for the peptides spanning the region 56–88 of HSA. The LVNEVTEF epitope is seen sliding through the overlapping 15-mer peptides. As part of the epitope leaves the peptide window (at position 67), the median native signal drops to 255 and new epitope residues are shown to be part of the epitope for the 15-mer VNEVTEFAKTCVADE. Some inconsistencies are found among the identified amino acids in overlapping peptides because these positions are only weakly affected by substitutions. Depending on the inclusion criterion the table shows a total of up to four overlapping epitopes. The results presented are nearly identical to previous studies made on the same data, using ANOVA-protected Tukey honest-significant-difference procedure to identify key residues (on the 0.01 significance level) involved in the epitopes of individual peptides [15]. Only the weakly affected residues fluctuate to a minor degree between the two approaches. A full table for the entire HSA protein can be found in S1 Table.

**Table 1:** Example of target-specific positions of individual peptides. The table shows output of 33 overlapping peptides mapping the positions 56 to 88 of the HSA protein sequence. Positions identified as being important for binding (identified by the Dunnett’s test of complete single-amino acid substitutions at the p<0.0001 level) are highlighted whereas dashes indicate positions not involved in binding. The median signal of copies of the corresponding native peptide is shown.

Position	Peptide	Epitope	Native Binding Intensity (Median)
56	QQCPFEDHVKLVNEV	---------------	62
57	QCPFEDHVKLVNEVT	---------LVNEV-	156
58	CPFEDHVKLVNEVTE	--------LVNEV-E	183
59	PFEDHVKLVNEVTEF	-------LVNEVTEF	560
60	FEDHVKLVNEVTEFA	------LVNEVTEF-	584
61	EDHVKLVNEVTEFAK	-----LVNEVTEF--	522
62	DHVKLVNEVTEFAKT	----LVNEVTEF---	497
63	HVKLVNEVTEFAKTC	---LVNEVTEF----	441
64	VKLVNEVTEFAKTCV	--LVNEVTEF-----	494
65	KLVNEVTEFAKTCVA	-LVNEVTEF------	396
66	LVNEVTEFAKTCVAD	LVNEVTEF--T----	473
67	VNEVTEFAKTCVADE	----TEF-KTCV--E	255
68	NEVTEFAKTCVADES	----EF-KT-V-DE-	327
69	EVTEFAKTCVADESA	---EFAKT-V-DE--	328
70	VTEFAKTCVADESAE	--EF--T-V-DES--	328
71	TEFAKTCVADESAEN	-EF--T-V-DES-E-	287
72	EFAKTCVADESAENC	-----CV-DESAE-C	350
73	FAKTCVADESAENCD	----C-ADESAE-C-	315
74	AKTCVADESAENCDK	---C-ADESAE-C--	270
75	KTCVADESAENCDKS	--C-ADESAE-C---	284
76	TCVADESAENCDKSL	-C--DESAE-C----	331
77	CVADESAENCDKSLH	C-ADESAE-CD----	206
78	VADESAENCDKSLHT	--DESAE--D-----	151
79	ADESAENCDKSLHTL	-DESAE--D------	136
80	DESAENCDKSLHTLF	----E--DKSL-TLF	395
81	ESAENCDKSLHTLFG	------DKSL-TLF-	355
82	SAENCDKSLHTLFGD	--E--DKSL-TLF--	388
83	AENCDKSLHTLFGDK	----DKSL-TLF-D-	331
84	ENCDKSLHTLFGDKL	---DKSL-TLF-D--	287
85	NCDKSLHTLFGDKLC	--DKSL-TLF-D---	199
86	CDKSLHTLFGDKLCT	-DKSL-TLF-D----	195
87	DKSLHTLFGDKLCTV	DKSL-TLF-D-----	174
88	KSLHTLFGDKLCTVA	---------------	44

### Antibody selectivity toward individual residues

**Fig 4:**
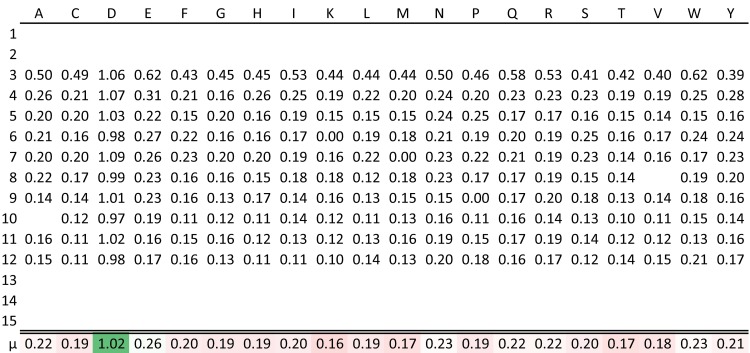
Example of selectivity tables. Tables showing substitution values of residue 518D of human serum albumin against all 20 amino acids (columns) and being represented in 10 out of 15 positions of the overlapping peptides (rows). Entire blank rows depict the native residue being represented in peptides with no positive signal. Individual blank cells are missing data. The row *μ* shows the mean substitution value of the replacing amino acids. Here, cells are highlighted by the effect of substitution as a color gradient from red to green through white, where white corresponds to the global mean, *μ*_*g*_, equal to 0.240.

The table output above presents a visual overview of the epitope mapping in overlapping peptides and enables the user to observe where one epitope ends and another begins. The output however, does not elucidate which positions are more selective than others for the antibody binding. To extract these differences, the algorithm incorporates the substitution values of a single protein residue from different peptides (native and 19 substitutions) overlapping this position. Such a substitution matrix is shown for position 518D in Fig 4, highlighting in the lower row the amino acid replacements with higher (green) or lower (red) substitution values relative to the global mean, *μ*_*g*_ = 0.240 (white), of the substitution matrix. Blank rows depict the native residue being represented in peptides with residues found to be significantly affected by substitution. The matrix displays complete selectivity for the native amino acid D (*μ*_*D*_ ≫ *μ*_*g*_), with all 19 amino acid variations disrupting antibody binding in the majority of positions being represented.

**Fig 5:**
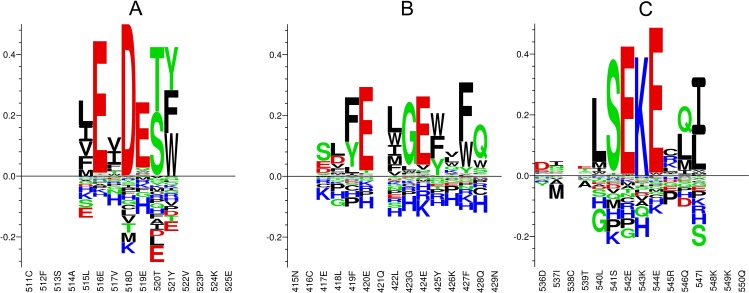
Examples of selectivity profile of epitope. Logo-plot representations of the selectivity of three 15-residue regions of the primary sequence of HSA. Selectivity is determined by Dunnett’s test on all substitutions of target position, being represented in overlapping peptides. Each letter represents replacing amino acids and is scaled by log(*p*), where the p-value is obtained from the Dunnett’s test. Positive letters denotes substitution values above the global mean, *μ*_*g*_, while negative letters denotes substitution values below *μ*_*g*_. The absolute height of an entire column represents the average relative change in signal of all substitutions of target residue. The average signal of native 15-mer peptides containing the epitopes is (**A**) 489 Au, (**B**) 130 Au and (**C**) 176 Au.

In order to visualize the selectivity, the algorithm constructs logo plots covering all positions in the target protein. As an example, the logo plot representation of three epitope regions of HSA (including residues 518D and 520T) is shown in Fig 5. A logo plot of the LEVDETY epitope is shown in Fig 5A. The figure shows amino acid letters in columns representing positions in the protein. Positions not previously found (using the single peptide analysis) to be significantly important for the epitope are shown as blank. The individual letters are log(*p*) scaled with positive letters denoting *μ*_*i*_ > *μ*_*g*_ and negative letters denoting *μ*_*i*_ < *μ*_*g*_. The absolute height of each position reflects the mean change (1 − *μ*_*g*_) caused by substitutions of the native amino acid. More details on the calculation of the logo plot and calculation of the position specific substitution matrices can be found in the methods section. The selectivity logo-plot shows a strong selectivity in positions 516E, 518D and 519E towards the native negatively charged amino acids while showing preference for non-polar residues in position 515L and 517V, small alcohol-containing residues (Serine and Threonine) in position 520T, and aromatic residues in position 521Y. Moreover, differences in the effect on substitutions of the native residues can be seen from the absolute height of letters in the logo-plot. Fig 5B and 5C shows examples of two other epitopes (ELFE-LGEYKFQ and DI-TLSEKERQI) found within peptides with relatively low binding signal (130 and 176 Au, respectively). A large number of single-amino acid derivatives of the ELFE-LGEYKFQ epitope share the binding signal of the native epitope, whereas only a few derivatives of the DI-TLSEKERQI epitope retain antibody binding. The results show that epitopes giving rise to similar binding signal may employ different effects upon substitutions (total height of columns in logo plot), and different representations of the amino acid selectivity. A full logo plot of the entire HSA protein can be found in S1 Fig.

The algorithm identifies a total of 18 HSA epitope regions, as shown in Table 2. Each identified position has at least one amino acid substitution leading to a significant mean relative change in signal. The selectivity of the individual positions differs greatly, which can be seen from the full logo plot of HSA in S1 Fig. Some regions span more than the 15-residue length of the peptides, and represents overlapping independent epitopes. The boundaries of individual epitopes are open to subjective evaluation by studying the individual overlapping peptides in S1 Table, and are not defined automatically by the algorithm.

**Table 2:** HSA epitopes. Table showing 18 HSA epitope regions identified by the algorithm. Dashes mark gaps of residues with no selectivity. The regions were identified from the logo plots, and defined as having a minimum of 4 residues and a maximum gap-length of 2 residues.

Range	Region
35–45	FKDLGEEN--A
66–96	LVNEVTEF-KTCVADESAE-CDKSL-TLF-D
101–106	VAT-RE
110–124	E--DCCAKQEPE--E
133–145	NPNLPRLV-RPEVD
151–158	F-DNEETF
176–184	PE-LFF-KR
203–209	LP-LDEL
229–235	KFGER-F
246–263	RFPKAEFAEVSKLV--LT
320–338	DEMPADLP-L-ADFVE-KD
398–408	FDEFKP--EEP
419–428	FE-LGEY-FQ
449–455	EVSRNLG
515–521	LEVDETY
540–547	LSEKE-QI
566–575	E-LKA--DDF
586–595	DD-ETC--EE

Two B cell epitopes for HSA have been mapped and are available within the IEDB [18]. Both of these are structural epitopes characterized with a large linear determinants; (E251, F252, A253, E254, S256, K257) and (S513, A514, L515, E516, E519, T520). Both of these are accurately captured by the ArrayPitope analysis of the peptide-chip data (see Table 2).

The application was applied to a library of complete single-amino acid substitutions of a native protein antigen sequences. Amino acid substitutions are a critical requirement of this algorithm. However, a single-residue scan, such as an alanine scan is sufficient to produce meaningful sequence logos that can be used to pinpoint non-alanine positions involved in the epitope. In such case, the substitution matrix in Fig 4 will contain only two columns, and the Dunnett’s statistical procedure will condense to a Student’s t-test of comparing the mean substitution value of the replacing amino acid with that of the native amino acid. The sliding representation of the epitopes (Table 1) will not be produced for this type of data, since the PSSM of Fig 3 would only include one substitution value for every position, critically lowering the degrees of freedom used for the Dunnett’s multiple comparison procedure. 13 epitope regions were found using a limited dataset including only substitutions with alanine (S2 Table). The alanine-only dataset was sufficient to identify 105 (55.9%) out of the 188 epitope residues found using the exhaustive substitution dataset (data not shown). 11 (10.5%) of the missed epitope positions can be ascribed to the native residue being alanine. The remaining missed epitope residues can be ascribed to a general limitation in epitope mapping when solely using alanine substitutions thus highlighting the value of a complete, exhaustive substitution strategy.

The algorithm has been made available as a webserver. It takes quantitative peptide data of fully or partially substituted overlapping peptides as well as protein sequence(s) in FASTA-format as input. Furthermore, options for specifying a custom significance level and length of peptides to be analyzed are available. The webserver outputs a table of overlapping peptides, each with their identified epitope residues highlighted, similar to Table 1, as well as sequence logos illustrating the specificity of the antibody-epitope complexes; see Fig 5 for details.

## Discussion

Insight into antibody-specificity of the binding sites (epitopes) of target proteins is important for the identification and design of diagnostic targets as well as characterization of therapeutic antibodies. Through the ability to express large numbers of peptides, the recent advances in high-density peptide microarrays facilitate high-throughput discovery of linear antibody epitopes. The large number of results calls for an automated method for analysing antibody-specificity. Here, we present a statistical approach to analyze antibody-specificity of epitopes from peptide-based single amino acid substitution data.

The pipeline is fully automated and consist of three main steps: i) mapping of peptides to the original target, ii) mapping of epitope positions of individual peptides by determining the statistical significance of substitutions and iii) determine the selectivity of each target residue involved in the epitope and visualize this by sequence logos. Using peptide microarray data containing a complete substitution analysis of HSA the tool was used to identify and characterize the specificity of 18 linear epitope regions of polyclonal rabbit anti-HSA antibodies.

The high-resolution substitution analysis allows the user to distinguish close-proximity epitopes of polyclonal antibodies, by viewing the epitope mapping in overlapping peptides sliding through the protein sequence analyzed, as exemplified in Table 1. Here, we illustrate how the method can deconvolute the presence of four individual antibody epitopes; when part of the high-signal epitope LVNEVTEF, starting at position 66, leaves the query peptide, the weaker, but overlapping epitope TEF-KT-V—E is revealed. When the N-terminal of this epitope leaves the query peptide window another overlapping epitope C-ADESAE-C appears, and yet another after this one. Identifying these epitopes solely from a signal profile, where only the signals of entire 15-mers are known, would be a challenge.

As thoroughly discussed by Buus et al. [10] the signal intensity is determined by a number of factors other than antibody affinity, such as peptide purity, solvation and antibody concentration. Moreover, it is often assumed that high-affinity antibodies are likely to be more specific, but affinity and specificity are not necessarily linked, as thoroughly discussed by Regenmortel [1]. Selecting epitope candidates purely on the basis of microarray binding signal may lead to relevant candidates bring discarded. As a demonstration, the epitope LSEKERQI, spanning the 540–547 region of HSA are presented in multiple peptides with a relatively weak signal of (114–170 Au), but the antibody specificity towards this epitope is comparable the that of the epitope LEVDETY in the signal range of 500 Au, see Fig 5.

When addressing specificity a word of caution is appropriate. The six CDRs of an antibody harbour multiple overlapping paratopes of 10–20 amino acids, and for an antibody to be monospecific to a single epitope it would require that the remaining part of the CDRs are unable to bind any other antigenic structure, which is unlikely [1]. The antibody may appear monospecific, however, only when tested for their capacity to bind one antigen and not another. Characterizing the specificity of antibody-epitope interaction by the degree of stereochemical complementarity upon single-amino acid substitutions may assist the selection of antibodies from polyclonal samples and understanding of the interaction of monoclonal antibodies.

There is considerable difference in selectivity between individual residues of the epitopes. Most epitopes identified here, contain a core of 3–4 highly selective residues and few residues where a certain chemical property is preferred. This information may prove crucial when characterizing potential cross reactivity to related targets. The logo-plots from the algorithm serve as a visual representation of the selectivity of individual epitope residues. Here, they have been used to identify the boundaries of epitope regions in the protein, as seen in Table 2.

One may find a few inconsistencies between the epitopes identified from individual peptides in Table 1 and the interpretation of selectivity by the logo-plots. A word of caution is hence appropriate, since the two analyses differ in representation of substitutions. While the PSSM of individual peptides is used to infer peptide positions significantly affected by substitutions, the substitutions of one position (column in the PSSM) with different amino acids may vary greatly, depending on the physiochemical properties of the replacing amino acid. As such, the different substitutions in the same peptide position may contribute a higher variance (and thus higher LSD) to the analysis, than multiple copies of the same replacing amino acid, consequently leading to false-negative epitope-positions. In the statistical analyses forming the basis of the logo-plots the pooled variance is calculated from copies of the same replacing amino acid, but represented in different peptide positions. Here, bias occurs when one protein position is represented in two overlapping epitopes, interacting with different antibodies. We strongly suggest that the two analyses outputs should be used in complement to each other.

In conclusion, we have presented an online analysis tool for automated characterization and visualization of antibody selectivity toward specificity-determining epitope residues from peptide microarray-driven substitution analysis. Although the method was developed with the aim of characterizing antibody-peptide interactions, it is not restricted to such interactions, but can readily analyse quantitative peptide data from any receptor-ligand interaction, provided that single-amino acid derivative peptides of the ligand peptides exist. We expect this application to be useful in complete substitution analysis of pre-identified binding peptides from multiple proteins, such as complete linear antibody epitope mapping of entire proteomes.

## Supporting Information

S1 Fig Selectivity profile of HSA epitopes. Logo-plot representations of the selectivity of epitope regions of HSA. Selectivity is determined by Dunnett’s test on all substitutions of target position, being represented in overlapping peptides. Each letter represents replacing amino acids and is scaled by log(*p*), where the p-value is obtained from the Dunnett’s test. Positive letters denote substitution values above the global mean, *μ*_*g*_, while negative letters denotes substitution values below *μ*_*g*_. The absolute height of an entire column represents the average relative change in signal of all substitutions of target residue.
(DOC)

S1 Table Target-specific positions of individual peptides. The table shows output of all overlapping peptides mapping the HSA protein sequence. Positions identified as being important for binding (identified by the Dunnett’s test of complete single-amino acid substitutions at the p<0.0001 level) are highlighted whereas dashes indicate positions not involved in binding. The median signal of copies of the corresponding native peptide is shown.
(DOC)

S2 Table HSA epitopes found through substitution with alanine only. Table showing 12 HSA epitope regions identified by the algorithm. The dataset was limited to only include alanine substitutions of native peptides of HSA. Dashes mark gaps of residues with no selectivity. The regions were identified from the logo plots (not shown), and defined as having a minimum of 4 residues and a maximum gap-length of 2 residues.
(DOC)
