# Supplementary material for: ArrayPitope: Automated Analysis of Amino Acid Substitutions for Peptide Microarray-Based Antibody Epitope Mapping
Source: PLoS One. 2017 Jan 17;12(1):e0168453. doi: 10.1371/journal.pone.0168453 (PMC5240915; doi:10.1371/journal.pone.0168453)
Supplement: S1 Fig — Logo-plot representations of the selectivity of epitope regions of HSA. Selectivity is determined by Dunnett’s test on all substitutions of target position, being represented in overlapping peptides. Each letter represents replacing amino acids and is scaled by log(p), where the p-value is obtained from the Dunnett’s test. Positive letters denote substitution values above the global mean, μg, while negative letters denotes substitution values below μg. The absolute height of an entire column represents the average relative change in signal of all substitutions of target residue. (DOC) [file pone.0168453.s001.doc]

**
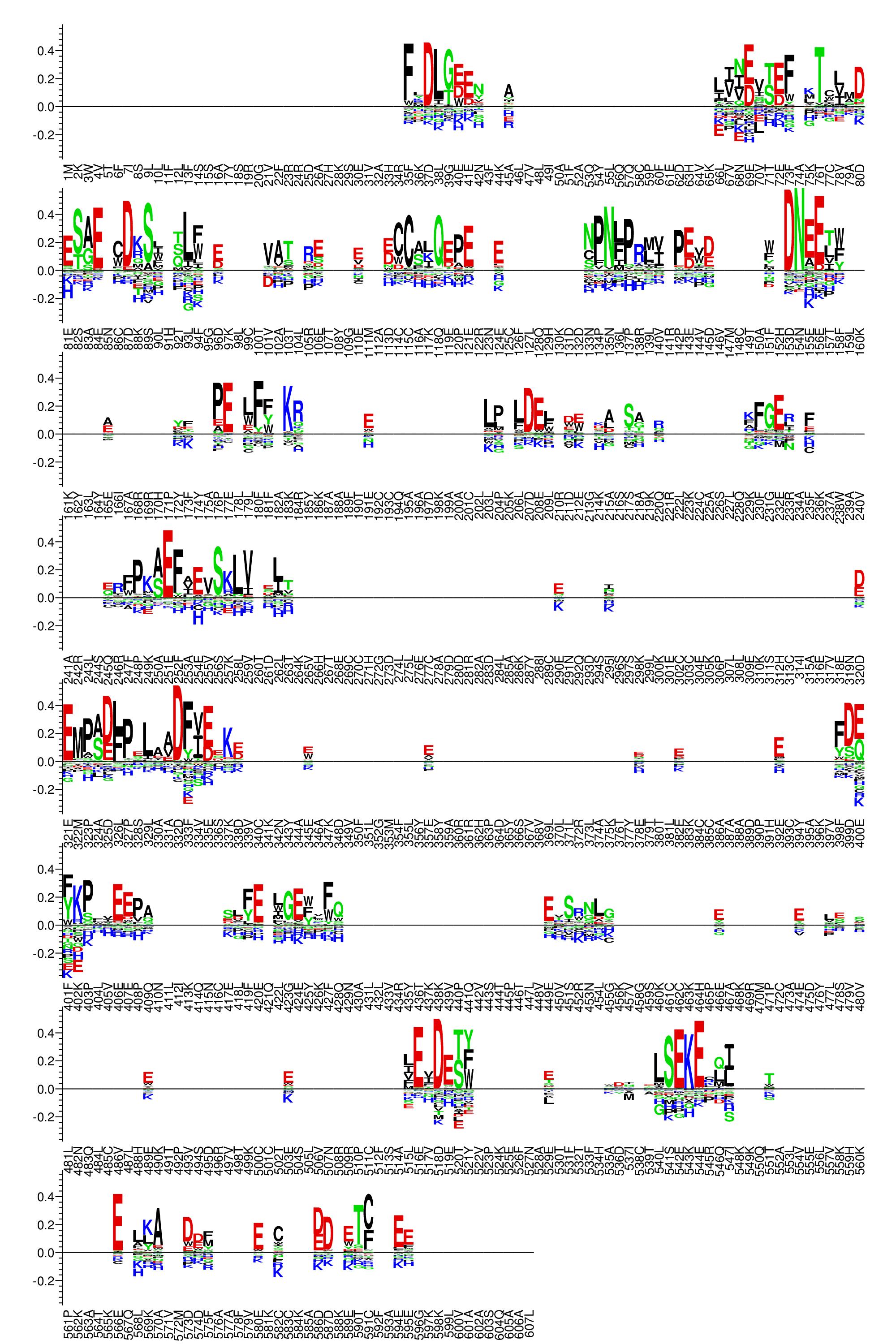
**

**S1 Figure. Selectivity profile of HSA epitopes.** Logo-plot representations of the selectivity of epitope regions of HSA. Selectivity is determined by Dunnett’s test on all substitutions of target position, being represented in overlapping peptides. Each letter represents replacing amino acids and is scaled by log(*p*), where the p-value is obtained from the Dunnett’s test. Positive letters denote substitution values above the global mean, *µ_g_*, while negative letters denotes substitution values below *µ_g_*. The absolute height of an entire column represents the average relative change in signal of all substitutions of target residue.
