# Supplementary material for: ArrayPitope: Automated Analysis of Amino Acid Substitutions for Peptide Microarray-Based Antibody Epitope Mapping
Source: PLoS One. 2017 Jan 17;12(1):e0168453. doi: 10.1371/journal.pone.0168453 (PMC5240915; doi:10.1371/journal.pone.0168453)
Supplement: S1 Table — The table shows output of all overlapping peptides mapping the HSA protein sequence. Positions identified as being important for binding (identified by the Dunnett’s test of complete single-amino acid substitutions at the p<0.0001 level) are highlighted whereas dashes indicate positions not involved in binding. The median signal of copies of the corresponding native peptide is shown. (DOC) [file pone.0168453.s002.doc]

**ArrayPitope: Automated analysis of amino acid substitutions for peptide microarray-based antibody epitope mapping**

Christian Skjødt Hansen^1^, Thomas Østerbye^2^, Paolo Marcatili^1^, Ole Lund^1^, Søren Buus^2^, and Morten Nielsen^1,3*^

^1^ Center for Biological Sequence Analysis, Department of Bio and Health Informatics, Technical University of Denmark, Kgs. Lyngby, Denmark

^2^ Laboratory of Experimental Immunology, Faculty of Health Sciences, University of Copenhagen, Copenhagen, Denmark

^3^ Instituto de Investigaciones Biotecnológicas, Universidad Nacional de San Martín, Buenos Aires, Argentina

* Corresponding author: Morten Nielsen, E-mail: mniel@cbs.dtu.dk

| Posi-tion | Peptide | Epitope | Native Signal |
| --- | --- | --- | --- |
| 1 | MKWVTFISLLFLFSS | --------------- | 55.50 |
| 2 | KWVTFISLLFLFSSA | ------------S-- | 55.50 |
| 3 | WVTFISLLFLFSSAY | --------------- | 59.00 |
| 4 | VTFISLLFLFSSAYS | --------------- | 58.00 |
| 5 | TFISLLFLFSSAYSR | --------------- | 60.50 |
| 6 | FISLLFLFSSAYSRG | --------------- | 52.00 |
| 7 | ISLLFLFSSAYSRGV | --------------- | 59.00 |
| 8 | SLLFLFSSAYSRGVF | --------------- | 64.50 |
| 9 | LLFLFSSAYSRGVFR | --------------- | 57.00 |
| 10 | LFLFSSAYSRGVFRR | --------------- | 64.00 |
| 11 | FLFSSAYSRGVFRRD | --------------- | 58.00 |
| 12 | LFSSAYSRGVFRRDA | --------------- | 58.00 |
| 13 | FSSAYSRGVFRRDAH | --------------- | 41.00 |
| 14 | SSAYSRGVFRRDAHK | --------------- | 35.00 |
| 15 | SAYSRGVFRRDAHKS | --------------- | 36.00 |
| 16 | AYSRGVFRRDAHKSE | --------------- | 34.00 |
| 17 | YSRGVFRRDAHKSEV | --------------- | 35.00 |
| 18 | SRGVFRRDAHKSEVA | --------------- | 37.00 |
| 19 | RGVFRRDAHKSEVAH | --------------- | 38.00 |
| 20 | GVFRRDAHKSEVAHR | --------------- | 35.00 |
| 21 | VFRRDAHKSEVAHRF | --------------- | 32.00 |
| 22 | FRRDAHKSEVAHRFK | --------------- | 28.00 |
| 23 | RRDAHKSEVAHRFKD | --------------- | 30.00 |
| 24 | RDAHKSEVAHRFKDL | --------------- | 38.00 |
| 25 | DAHKSEVAHRFKDLG | ----------F-DLG | 53.00 |
| 26 | AHKSEVAHRFKDLGE | ---------FKDLGE | 105.00 |
| 27 | HKSEVAHRFKDLGEE | --------FKDLGEE | 139.00 |
| 28 | KSEVAHRFKDLGEEN | -------FKDLGEE- | 150.00 |
| 29 | SEVAHRFKDLGEENF | ------FKDLGEE-- | 165.00 |
| 30 | EVAHRFKDLGEENFK | -----F-DLGEE--- | 156.00 |
| 31 | VAHRFKDLGEENFKA | ----F-DLGEE---- | 137.00 |
| 32 | AHRFKDLGEENFKAL | ---F-DLGEE----- | 116.00 |
| Posi-tion | Peptide | Epitope | Native Signal |
| 33 | HRFKDLGEENFKALV | --FKDLGEE------ | 114.00 |
| 34 | RFKDLGEENFKALVL | -F-DLGEE------- | 120.00 |
| 35 | FKDLGEENFKALVLI | F-DLGEE-------- | 128.00 |
| 36 | KDLGEENFKALVLIA | -D--EEN--AL-LI- | 81.00 |
| 37 | DLGEENFKALVLIAF | ---EEN--A------ | 96.00 |
| 38 | LGEENFKALVLIAFA | ---E----------- | 81.00 |
| 39 | GEENFKALVLIAFAQ | --E------------ | 67.00 |
| 40 | EENFKALVLIAFAQY | --------------- | 61.00 |
| 41 | ENFKALVLIAFAQYL | --------------- | 57.00 |
| 42 | NFKALVLIAFAQYLQ | --------------- | 48.00 |
| 43 | FKALVLIAFAQYLQQ | --------------- | 43.00 |
| 44 | KALVLIAFAQYLQQC | --------------- | 46.00 |
| 45 | ALVLIAFAQYLQQCP | --------------- | 55.00 |
| 46 | LVLIAFAQYLQQCPF | --------------- | 54.00 |
| 47 | VLIAFAQYLQQCPFE | --------------- | 58.00 |
| 48 | LIAFAQYLQQCPFED | --------------- | 66.00 |
| 49 | IAFAQYLQQCPFEDH | --------------- | 61.00 |
| 50 | AFAQYLQQCPFEDHV | --------------- | 54.00 |
| 51 | FAQYLQQCPFEDHVK | --------------- | 58.00 |
| 52 | AQYLQQCPFEDHVKL | --------------- | 50.00 |
| 53 | QYLQQCPFEDHVKLV | --------------- | 44.00 |
| 54 | YLQQCPFEDHVKLVN | -------E------- | 59.00 |
| 55 | LQQCPFEDHVKLVNE | --------------- | 50.00 |
| 56 | QQCPFEDHVKLVNEV | --------------- | 62.00 |
| 57 | QCPFEDHVKLVNEVT | ---------LVNEV- | 156.00 |
| 58 | CPFEDHVKLVNEVTE | --------LVNEV-E | 183.00 |
| 59 | PFEDHVKLVNEVTEF | -------LVNEVTEF | 560.00 |
| 60 | FEDHVKLVNEVTEFA | ------LVNEVTEF- | 584.00 |
| 61 | EDHVKLVNEVTEFAK | -----LVNEVTEF-- | 522.00 |
| 62 | DHVKLVNEVTEFAKT | ----LVNEVTEF--- | 497.00 |
| 63 | HVKLVNEVTEFAKTC | ---LVNEVTEF---- | 441.00 |
| 64 | VKLVNEVTEFAKTCV | --LVNEVTEF----- | 494.00 |
| Posi-tion | Peptide | Epitope | Native Signal |
| 65 | KLVNEVTEFAKTCVA | -LVNEVTEF------ | 396.00 |
| 66 | LVNEVTEFAKTCVAD | LVNEVTEF--T---- | 473.00 |
| 67 | VNEVTEFAKTCVADE | ----TEF-KTCV--E | 255.00 |
| 68 | NEVTEFAKTCVADES | ----EF-KT-V-DE- | 327.00 |
| 69 | EVTEFAKTCVADESA | ---EFAKT-V-DE-- | 328.00 |
| 70 | VTEFAKTCVADESAE | --EF--T-V-DES-- | 328.00 |
| 71 | TEFAKTCVADESAEN | -EF--T-V-DES-E- | 287.00 |
| 72 | EFAKTCVADESAENC | -----CV-DESAE-C | 350.00 |
| 73 | FAKTCVADESAENCD | ----C-ADESAE-C- | 315.00 |
| 74 | AKTCVADESAENCDK | ---C-ADESAE-C-- | 270.00 |
| 75 | KTCVADESAENCDKS | --C-ADESAE-C--- | 284.00 |
| 76 | TCVADESAENCDKSL | -C--DESAE-C---- | 331.00 |
| 77 | CVADESAENCDKSLH | C-ADESAE-CD---- | 206.00 |
| 78 | VADESAENCDKSLHT | --DESAE--D----- | 151.00 |
| 79 | ADESAENCDKSLHTL | -DESAE--D------ | 136.00 |
| 80 | DESAENCDKSLHTLF | ----E--DKSL-TLF | 395.00 |
| 81 | ESAENCDKSLHTLFG | ------DKSL-TLF- | 355.00 |
| 82 | SAENCDKSLHTLFGD | --E--DKSL-TLF-- | 388.00 |
| 83 | AENCDKSLHTLFGDK | ----DKSL-TLF-D- | 331.00 |
| 84 | ENCDKSLHTLFGDKL | ---DKSL-TLF-D-- | 287.00 |
| 85 | NCDKSLHTLFGDKLC | --DKSL-TLF-D--- | 199.00 |
| 86 | CDKSLHTLFGDKLCT | -DKSL-TLF-D---- | 195.00 |
| 87 | DKSLHTLFGDKLCTV | DKSL-TLF-D----- | 174.00 |
| 88 | KSLHTLFGDKLCTVA | --------------- | 44.00 |
| 89 | SLHTLFGDKLCTVAT | --------------- | 51.00 |
| 90 | LHTLFGDKLCTVATL | --------------- | 53.00 |
| 91 | HTLFGDKLCTVATLR | --------------- | 56.00 |
| 92 | TLFGDKLCTVATLRE | --------------- | 74.00 |
| 93 | LFGDKLCTVATLRET | --------------- | 71.00 |
| 94 | FGDKLCTVATLRETY | --------AT--E-- | 88.00 |
| 95 | GDKLCTVATLRETYG | ------VAT-RE--- | 88.00 |
| 96 | DKLCTVATLRETYGE | -----VAT-RE---- | 117.00 |
| 97 | KLCTVATLRETYGEM | ----VAT-RE----- | 111.00 |
| 98 | LCTVATLRETYGEMA | ---VAT-RE------ | 103.00 |
| 99 | CTVATLRETYGEMAD | --VA----------- | 84.00 |
| 100 | TVATLRETYGEMADC | -VAT-RET------- | 98.00 |
| 101 | VATLRETYGEMADCC | --------------- | 97.00 |
| 102 | ATLRETYGEMADCCA | --------E------ | 78.00 |
| 103 | TLRETYGEMADCCAK | -------E------- | 71.00 |
| 104 | LRETYGEMADCCAKQ | ------E----C--- | 79.00 |
| 105 | RETYGEMADCCAKQE | --------DCC--Q- | 96.00 |
| 106 | ETYGEMADCCAKQEP | -------DCC--QE- | 115.00 |
| 107 | TYGEMADCCAKQEPE | ---E--DCC--QE-- | 137.00 |
| 108 | YGEMADCCAKQEPER | -----DCC--QE-E- | 137.00 |
| 109 | GEMADCCAKQEPERN | ----DCCAKQE-E-- | 167.00 |
| 110 | EMADCCAKQEPERNE | ---DCC--Q------ | 171.00 |
| 111 | MADCCAKQEPERNEC | -----AKQ--E---- | 125.00 |
| 112 | ADCCAKQEPERNECF | --C-AKQEPE----- | 189.00 |
| 113 | DCCAKQEPERNECFL | -----Q-PE------ | 132.00 |
| 114 | CCAKQEPERNECFLQ | ----Q-PE--E---- | 134.00 |
| 115 | CAKQEPERNECFLQH | ---Q-PE--E----- | 70.00 |
| 116 | AKQEPERNECFLQHK | -----E--E------ | 52.00 |
| 117 | KQEPERNECFLQHKD | --------------- | 48.00 |
| 118 | QEPERNECFLQHKDD | --------------- | 51.00 |
| 119 | EPERNECFLQHKDDN | --------------- | 46.00 |
| 120 | PERNECFLQHKDDNP | --------------- | 46.00 |
| 121 | ERNECFLQHKDDNPN | --------------- | 47.00 |
| 122 | RNECFLQHKDDNPNL | --------------- | 58.00 |
| 123 | NECFLQHKDDNPNLP | --------D-NPNL- | 99.00 |
| 124 | ECFLQHKDDNPNLPR | ---------NPN--- | 165.00 |
| Posi-tion | Peptide | Epitope | Native Signal |
| 125 | CFLQHKDDNPNLPRL | ---------PNLP-- | 219.00 |
| 126 | FLQHKDDNPNLPRLV | ---------NLP--- | 288.00 |
| 127 | LQHKDDNPNLPRLVR | --------NLP---- | 275.00 |
| 128 | QHKDDNPNLPRLVRP | -------N------- | 215.00 |
| 129 | HKDDNPNLPRLVRPE | --------------- | 200.00 |
| 130 | KDDNPNLPRLVRPEV | -----NL-------- | 232.00 |
| 131 | DDNPNLPRLVRPEVD | ----NLP-------- | 323.00 |
| 132 | DNPNLPRLVRPEVDV | ---NLPRLV------ | 207.00 |
| 133 | NPNLPRLVRPEVDVM | ---LP---------- | 140.00 |
| 134 | PNLPRLVRPEVDVMC | ----RLV-PE----- | 138.00 |
| 135 | NLPRLVRPEVDVMCT | ----LV-PE------ | 106.00 |
| 136 | LPRLVRPEVDVMCTA | ---LV-PE------- | 83.00 |
| 137 | PRLVRPEVDVMCTAF | -----PEVD------ | 91.00 |
| 138 | RLVRPEVDVMCTAFH | -----EVD------- | 66.00 |
| 139 | LVRPEVDVMCTAFHD | ----EVD-------- | 76.00 |
| 140 | VRPEVDVMCTAFHDN | --------------- | 58.00 |
| 141 | RPEVDVMCTAFHDNE | --------------- | 65.00 |
| 142 | PEVDVMCTAFHDNEE | --------------- | 59.00 |
| 143 | EVDVMCTAFHDNEET | --------------- | 77.00 |
| 144 | VDVMCTAFHDNEETF | -------F-DNEETF | 167.00 |
| 145 | DVMCTAFHDNEETFL | ------F-DNEETF- | 260.00 |
| 146 | VMCTAFHDNEETFLK | -----F-DNEETF-- | 342.00 |
| 147 | MCTAFHDNEETFLKK | ------DNEETF--- | 419.00 |
| 148 | CTAFHDNEETFLKKY | ---F-DNEETF---- | 497.00 |
| 149 | TAFHDNEETFLKKYL | --F-DNEETF----- | 556.00 |
| 150 | AFHDNEETFLKKYLY | ---DNEETF------ | 410.50 |
| 151 | FHDNEETFLKKYLYE | --DNEETF------- | 331.00 |
| 152 | HDNEETFLKKYLYEI | -DNEETF-------- | 121.00 |
| 153 | DNEETFLKKYLYEIA | DNEETFL-------- | 147.00 |
| 154 | NEETFLKKYLYEIAR | --------------- | 71.00 |
| 155 | EETFLKKYLYEIARR | --------------- | 59.00 |
| 156 | ETFLKKYLYEIARRH | --------------- | 44.00 |
| 157 | TFLKKYLYEIARRHP | --------E------ | 39.00 |
| 158 | FLKKYLYEIARRHPY | F-------------- | 40.00 |
| 159 | LKKYLYEIARRHPYF | --------------- | 34.00 |
| 160 | KKYLYEIARRHPYFY | --------------- | 22.00 |
| 161 | KYLYEIARRHPYFYA | --------------- | 25.00 |
| 162 | YLYEIARRHPYFYAP | YLYE-A----YFYA- | 43.50 |
| 163 | LYEIARRHPYFYAPE | --------------- | 40.50 |
| 164 | YEIARRHPYFYAPEL | -------------E- | 39.50 |
| 165 | EIARRHPYFYAPELL | -------Y------- | 44.50 |
| 166 | IARRHPYFYAPELLF | -----------E--- | 42.00 |
| 167 | ARRHPYFYAPELLFF | ----------E---- | 44.50 |
| 168 | RRHPYFYAPELLFFA | -----F---E--FF- | 42.00 |
| 169 | RHPYFYAPELLFFAK | --------E--FF-- | 45.00 |
| 170 | HPYFYAPELLFFAKR | ------PE--FF-KR | 112.00 |
| 171 | PYFYAPELLFFAKRY | -----PE--FF-KR- | 110.00 |
| 172 | YFYAPELLFFAKRYK | ----PE--FF-K--- | 72.00 |
| 173 | FYAPELLFFAKRYKA | ---PE--FF-K---- | 66.00 |
| 174 | YAPELLFFAKRYKAA | --PE-LF--KR---- | 50.00 |
| 175 | APELLFFAKRYKAAF | -PE-LF--K------ | 48.00 |
| 176 | PELLFFAKRYKAAFT | PE-LF--K------- | 97.00 |
| 177 | ELLFFAKRYKAAFTE | E--F----------- | 40.00 |
| 178 | LLFFAKRYKAAFTEC | --------------- | 29.00 |
| 179 | LFFAKRYKAAFTECC | ------------E-- | 32.00 |
| 180 | FFAKRYKAAFTECCQ | -----------E--- | 32.00 |
| 181 | FAKRYKAAFTECCQA | ----------E---- | 26.00 |
| 182 | AKRYKAAFTECCQAA | --------------- | 25.00 |
| 183 | KRYKAAFTECCQAAD | --------------- | 22.00 |
| 184 | RYKAAFTECCQAADK | --------------- | 26.00 |
| Posi-tion | Peptide | Epitope | Native Signal |
| 185 | YKAAFTECCQAADKA | --------------- | 31.00 |
| 186 | KAAFTECCQAADKAA | --------------- | 28.50 |
| 187 | AAFTECCQAADKAAC | ----E---------- | 37.00 |
| 188 | AFTECCQAADKAACL | ---E-----D---C- | 49.00 |
| 189 | FTECCQAADKAACLL | --E------------ | 43.00 |
| 190 | TECCQAADKAACLLP | -E------------- | 36.00 |
| 191 | ECCQAADKAACLLPK | --------------- | 24.00 |
| 192 | CCQAADKAACLLPKL | --------------- | 21.00 |
| 193 | CQAADKAACLLPKLD | --------------- | 29.00 |
| 194 | QAADKAACLLPKLDE | --------------E | 41.50 |
| 195 | AADKAACLLPKLDEL | --------LP-LDE- | 61.00 |
| 196 | ADKAACLLPKLDELR | -------L--LDE-- | 62.00 |
| 197 | DKAACLLPKLDELRD | -----LLP-LDEL-- | 73.00 |
| 198 | KAACLLPKLDELRDE | --------LDE---- | 52.00 |
| 199 | AACLLPKLDELRDEG | -------LDE----- | 63.50 |
| 200 | ACLLPKLDELRDEGK | ------LDEL--E-- | 62.00 |
| 201 | CLLPKLDELRDEGKA | -----LDE---E--- | 59.00 |
| 202 | LLPKLDELRDEGKAS | -----DE---E---- | 57.00 |
| 203 | LPKLDELRDEGKASS | ---LDEL--E----- | 58.00 |
| 204 | PKLDELRDEGKASSA | --------------- | 38.00 |
| 205 | KLDELRDEGKASSAK | --------------- | 27.00 |
| 206 | LDELRDEGKASSAKQ | -----DE--A-SA-- | 50.00 |
| 207 | DELRDEGKASSAKQR | -----E--A-SA--- | 39.50 |
| 208 | ELRDEGKASSAKQRL | E--DE--A-SA-Q-- | 37.00 |
| 209 | LRDEGKASSAKQRLK | -----K--------- | 29.00 |
| 210 | RDEGKASSAKQRLKC | -----A-S------- | 23.00 |
| 211 | DEGKASSAKQRLKCA | -------A------- | 22.00 |
| 212 | EGKASSAKQRLKCAS | --K-----Q------ | 23.00 |
| 213 | GKASSAKQRLKCASL | --------------- | 32.00 |
| 214 | KASSAKQRLKCASLQ | --------------- | 29.00 |
| 215 | ASSAKQRLKCASLQK | --------------- | 37.00 |
| 216 | SSAKQRLKCASLQKF | --------------- | 34.00 |
| 217 | SAKQRLKCASLQKFG | --------------- | 37.00 |
| 218 | AKQRLKCASLQKFGE | --------------- | 38.00 |
| 219 | KQRLKCASLQKFGER | --------------- | 35.00 |
| 220 | QRLKCASLQKFGERA | ------------E-- | 47.00 |
| 221 | RLKCASLQKFGERAF | ---------FGER-F | 79.00 |
| 222 | LKCASLQKFGERAFK | -------KFGER-F- | 73.00 |
| 223 | KCASLQKFGERAFKA | ------KFGER---- | 64.00 |
| 224 | CASLQKFGERAFKAW | ------FGER-F--- | 80.00 |
| 225 | ASLQKFGERAFKAWA | -----FGE--F---- | 76.00 |
| 226 | SLQKFGERAFKAWAV | ----F-E--F----- | 70.00 |
| 227 | LQKFGERAFKAWAVA | ---FGE--F------ | 71.00 |
| 228 | QKFGERAFKAWAVAR | --------------- | 54.00 |
| 229 | KFGERAFKAWAVARL | -FGE--F-------- | 69.00 |
| 230 | FGERAFKAWAVARLS | --------------- | 57.00 |
| 231 | GERAFKAWAVARLSQ | --------------- | 55.00 |
| 232 | ERAFKAWAVARLSQR | --------------- | 46.00 |
| 233 | RAFKAWAVARLSQRF | --------------- | 48.00 |
| 234 | AFKAWAVARLSQRFP | --------------- | 52.00 |
| 235 | FKAWAVARLSQRFPK | --------------- | 44.00 |
| 236 | KAWAVARLSQRFPKA | ---------QRFP-A | 62.00 |
| 237 | AWAVARLSQRFPKAE | --------------- | 66.00 |
| 238 | WAVARLSQRFPKAEF | -------Q-FP-AE- | 83.00 |
| 239 | AVARLSQRFPKAEFA | --------FPKAEF- | 124.00 |
| 240 | VARLSQRFPKAEFAE | -----QRFPKAEFAE | 166.00 |
| 241 | ARLSQRFPKAEFAEV | ----QRFPKAEFAE- | 176.00 |
| 242 | RLSQRFPKAEFAEVS | -----FPKAEFAE-- | 183.00 |
| 243 | LSQRFPKAEFAEVSK | ----FPKAEFAE--- | 186.00 |
| 244 | SQRFPKAEFAEVSKL | ---FPKAEFAE---- | 163.00 |
| Posi-tion | Peptide | Epitope | Native Signal |
| 245 | QRFPKAEFAEVSKLV | ---PKAEFAE----- | 153.00 |
| 246 | RFPKAEFAEVSKLVT | -FPKAEFAE------ | 139.00 |
| 247 | FPKAEFAEVSKLVTD | -PKAEFAE------- | 138.00 |
| 248 | PKAEFAEVSKLVTDL | P-AEFAEVSKLV--L | 180.00 |
| 249 | KAEFAEVSKLVTDLT | --EF-EVSKLV--LT | 174.00 |
| 250 | AEFAEVSKLVTDLTK | ----EVSKLV--LT- | 193.00 |
| 251 | EFAEVSKLVTDLTKV | ---EVSKLV-DLT-- | 160.00 |
| 252 | FAEVSKLVTDLTKVH | --EVSKLVTDLT--- | 121.00 |
| 253 | AEVSKLVTDLTKVHT | -EVSKLV--LT---- | 109.00 |
| 254 | EVSKLVTDLTKVHTE | EVSKLV--LT----E | 127.00 |
| 255 | VSKLVTDLTKVHTEC | ----V---------- | 59.00 |
| 256 | SKLVTDLTKVHTECC | --------------- | 52.00 |
| 257 | KLVTDLTKVHTECCH | --------------- | 43.00 |
| 258 | LVTDLTKVHTECCHG | --------------- | 43.00 |
| 259 | VTDLTKVHTECCHGD | --------------- | 50.00 |
| 260 | TDLTKVHTECCHGDL | --------------- | 44.00 |
| 261 | DLTKVHTECCHGDLL | --------------- | 46.00 |
| 262 | LTKVHTECCHGDLLE | --------------- | 50.00 |
| 263 | TKVHTECCHGDLLEC | --------------- | 51.00 |
| 264 | KVHTECCHGDLLECA | --------------- | 51.00 |
| 265 | VHTECCHGDLLECAD | --------------- | 56.00 |
| 266 | HTECCHGDLLECADD | --------------- | 59.00 |
| 267 | TECCHGDLLECADDR | --------------- | 57.00 |
| 268 | ECCHGDLLECADDRA | --------------- | 61.00 |
| 269 | CCHGDLLECADDRAD | --------------- | 56.00 |
| 270 | CHGDLLECADDRADL | --------------- | 61.00 |
| 271 | HGDLLECADDRADLA | --------------- | 62.00 |
| 272 | GDLLECADDRADLAK | --------------- | 64.00 |
| 273 | DLLECADDRADLAKY | --------------- | 62.00 |
| 274 | LLECADDRADLAKYI | --------------- | 62.00 |
| 275 | LECADDRADLAKYIC | --------------- | 64.00 |
| 276 | ECADDRADLAKYICE | --------------- | 63.00 |
| 277 | CADDRADLAKYICEN | --------------- | 68.00 |
| 278 | ADDRADLAKYICENQ | --------------- | 72.00 |
| 279 | DDRADLAKYICENQD | -----L--YI-E--- | 77.00 |
| 280 | DRADLAKYICENQDS | ----------E---- | 73.00 |
| 281 | RADLAKYICENQDSI | ---------E----- | 69.00 |
| 282 | ADLAKYICENQDSIS | --------E------ | 69.00 |
| 283 | DLAKYICENQDSISS | -------E------- | 81.00 |
| 284 | LAKYICENQDSISSK | --------------- | 75.00 |
| 285 | AKYICENQDSISSKL | --------------- | 68.00 |
| 286 | KYICENQDSISSKLK | --------------- | 59.00 |
| 287 | YICENQDSISSKLKE | --------IS----- | 85.00 |
| 288 | ICENQDSISSKLKEC | --------------- | 79.00 |
| 289 | CENQDSISSKLKECC | ------I-----E-- | 78.00 |
| 290 | ENQDSISSKLKECCE | --------------- | 73.00 |
| 291 | NQDSISSKLKECCEK | --------------- | 68.00 |
| 292 | QDSISSKLKECCEKP | --------------- | 65.00 |
| 293 | DSISSKLKECCEKPL | --------------- | 66.00 |
| 294 | SISSKLKECCEKPLL | ----------E---- | 61.00 |
| 295 | ISSKLKECCEKPLLE | --------------- | 59.00 |
| 296 | SSKLKECCEKPLLEK | --------------- | 51.00 |
| 297 | SKLKECCEKPLLEKS | --------------- | 57.00 |
| 298 | KLKECCEKPLLEKSH | --------------- | 50.00 |
| 299 | LKECCEKPLLEKSHC | --------------- | 61.00 |
| 300 | KECCEKPLLEKSHCI | --------------- | 52.00 |
| 301 | ECCEKPLLEKSHCIA | --------------- | 59.00 |
| 302 | CCEKPLLEKSHCIAE | --------------- | 53.00 |
| 303 | CEKPLLEKSHCIAEV | --------------- | 55.00 |
| 304 | EKPLLEKSHCIAEVE | --------------- | 60.00 |
| Posi-tion | Peptide | Epitope | Native Signal |
| 305 | KPLLEKSHCIAEVEN | --------------- | 54.00 |
| 306 | PLLEKSHCIAEVEND | --------------- | 63.00 |
| 307 | LLEKSHCIAEVENDE | --------------- | 56.00 |
| 308 | LEKSHCIAEVENDEM | --------------- | 60.00 |
| 309 | EKSHCIAEVENDEMP | --------------- | 56.00 |
| 310 | KSHCIAEVENDEMPA | ----------DEMPA | 114.00 |
| 311 | SHCIAEVENDEMPAD | ---------DEMPAD | 89.00 |
| 312 | HCIAEVENDEMPADL | --------DEMPADL | 192.00 |
| 313 | CIAEVENDEMPADLP | -------DEMPADLP | 297.00 |
| 314 | IAEVENDEMPADLPS | ------DEMPADLP- | 381.00 |
| 315 | AEVENDEMPADLPSL | -----DEMPADLP-- | 387.00 |
| 316 | EVENDEMPADLPSLA | ----DEMPADLPSL- | 458.00 |
| 317 | VENDEMPADLPSLAA | ----EMPADLP---- | 396.00 |
| 318 | ENDEMPADLPSLAAD | --DEMPADLP----- | 349.00 |
| 319 | NDEMPADLPSLAADF | -DEMPADLP-L--D- | 374.00 |
| 320 | DEMPADLPSLAADFV | DEMPADLPSLAADFV | 723.00 |
| 321 | EMPADLPSLAADFVE | -----L--LAADFVE | 521.00 |
| 322 | MPADLPSLAADFVES | ------SLAADFVES | 554.00 |
| 323 | PADLPSLAADFVESK | ------LAADFVESK | 636.00 |
| 324 | ADLPSLAADFVESKD | -----LAADFVE--- | 590.00 |
| 325 | DLPSLAADFVESKDV | ----LAADFVESK-- | 595.00 |
| 326 | LPSLAADFVESKDVC | ---L-ADFVESK--- | 603.00 |
| 327 | PSLAADFVESKDVCK | --L-ADFVESKD--- | 537.00 |
| 328 | SLAADFVESKDVCKN | -LAADFVES-D---- | 510.00 |
| 329 | LAADFVESKDVCKNY | L-ADFVE-KD----- | 423.00 |
| 330 | AADFVESKDVCKNYA | --DFVE-KD------ | 214.00 |
| 331 | ADFVESKDVCKNYAE | -DFVE-KD------E | 263.00 |
| 332 | DFVESKDVCKNYAEA | DFVE-KD-------- | 237.00 |
| 333 | FVESKDVCKNYAEAK | --E------------ | 66.00 |
| 334 | VESKDVCKNYAEAKD | --------------- | 60.00 |
| 335 | ESKDVCKNYAEAKDV | --------------- | 53.00 |
| 336 | SKDVCKNYAEAKDVF | --------------- | 49.00 |
| 337 | KDVCKNYAEAKDVFL | --------------- | 48.00 |
| 338 | DVCKNYAEAKDVFLG | --------------- | 60.00 |
| 339 | VCKNYAEAKDVFLGM | --------------- | 58.00 |
| 340 | CKNYAEAKDVFLGMF | --------------- | 62.00 |
| 341 | KNYAEAKDVFLGMFL | --------------- | 63.00 |
| 342 | NYAEAKDVFLGMFLY | --------------- | 71.00 |
| 343 | YAEAKDVFLGMFLYE | --E------------ | 77.00 |
| 344 | AEAKDVFLGMFLYEY | --------------- | 76.00 |
| 345 | EAKDVFLGMFLYEYA | ------------E-- | 74.00 |
| 346 | AKDVFLGMFLYEYAR | --------------- | 57.00 |
| 347 | KDVFLGMFLYEYARR | ----------E---- | 53.00 |
| 348 | DVFLGMFLYEYARRH | --------------- | 55.00 |
| 349 | VFLGMFLYEYARRHP | --------------- | 49.00 |
| 350 | FLGMFLYEYARRHPD | -------E------- | 49.00 |
| 351 | LGMFLYEYARRHPDY | --------------- | 52.00 |
| 352 | GMFLYEYARRHPDYS | --------------- | 52.00 |
| 353 | MFLYEYARRHPDYSV | --------------- | 50.00 |
| 354 | FLYEYARRHPDYSVV | --------------- | 55.00 |
| 355 | LYEYARRHPDYSVVL | --------------- | 53.00 |
| 356 | YEYARRHPDYSVVLL | --------------- | 50.00 |
| 357 | EYARRHPDYSVVLLL | --------------- | 51.00 |
| 358 | YARRHPDYSVVLLLR | --------------- | 45.00 |
| 359 | ARRHPDYSVVLLLRL | --------------- | 43.00 |
| 360 | RRHPDYSVVLLLRLA | --------------- | 43.00 |
| 361 | RHPDYSVVLLLRLAK | --------------- | 42.00 |
| 362 | HPDYSVVLLLRLAKT | --------------- | 44.00 |
| 363 | PDYSVVLLLRLAKTY | --------------- | 51.00 |
| 364 | DYSVVLLLRLAKTYE | --------------- | 56.00 |
| Posi-tion | Peptide | Epitope | Native Signal |
| 365 | YSVVLLLRLAKTYET | --------------- | 56.00 |
| 366 | SVVLLLRLAKTYETT | ------------E-- | 58.00 |
| 367 | VVLLLRLAKTYETTL | --------------- | 58.00 |
| 368 | VLLLRLAKTYETTLE | --------------- | 62.00 |
| 369 | LLLRLAKTYETTLEK | --------------- | 67.00 |
| 370 | LLRLAKTYETTLEKC | --------------- | 59.00 |
| 371 | LRLAKTYETTLEKCC | --------------- | 56.00 |
| 372 | RLAKTYETTLEKCCA | --------------- | 49.00 |
| 373 | LAKTYETTLEKCCAA | -----E---E----- | 58.00 |
| 374 | AKTYETTLEKCCAAA | --------------- | 49.00 |
| 375 | KTYETTLEKCCAAAD | --------------- | 54.00 |
| 376 | TYETTLEKCCAAADP | --------------- | 60.00 |
| 377 | YETTLEKCCAAADPH | --------------- | 49.00 |
| 378 | ETTLEKCCAAADPHE | --------------- | 47.00 |
| 379 | TTLEKCCAAADPHEC | --------------- | 56.00 |
| 380 | TLEKCCAAADPHECY | ------------E-- | 62.00 |
| 381 | LEKCCAAADPHECYA | -----------E--- | 61.00 |
| 382 | EKCCAAADPHECYAK | E-------P-E---- | 58.00 |
| 383 | KCCAAADPHECYAKV | ------D--E----- | 44.00 |
| 384 | CCAAADPHECYAKVF | ---A----E------ | 60.00 |
| 385 | CAAADPHECYAKVFD | --------------- | 54.00 |
| 386 | AAADPHECYAKVFDE | ------E-------- | 52.00 |
| 387 | AADPHECYAKVFDEF | --------------- | 57.00 |
| 388 | ADPHECYAKVFDEFK | ---------VFDEFK | 139.00 |
| 389 | DPHECYAKVFDEFKP | ---------FDEFKP | 231.00 |
| 390 | PHECYAKVFDEFKPL | --------FDEFKPL | 331.00 |
| 391 | HECYAKVFDEFKPLV | -------FDEFKP-V | 602.00 |
| 392 | ECYAKVFDEFKPLVE | -------DEFKP--E | 680.00 |
| 393 | CYAKVFDEFKPLVEE | ------DEFKP-VE- | 688.00 |
| 394 | YAKVFDEFKPLVEEP | -----DEFKP--E-- | 694.00 |
| 395 | AKVFDEFKPLVEEPQ | ----DEFKP--E--- | 655.00 |
| 396 | KVFDEFKPLVEEPQN | ---DEFKP--E---- | 675.00 |
| 397 | VFDEFKPLVEEPQNL | --DEFKP--E----- | 664.00 |
| 398 | FDEFKPLVEEPQNLI | -DEFKP--E------ | 601.00 |
| 399 | DEFKPLVEEPQNLIK | DEFKP-VEE------ | 583.00 |
| 400 | EFKPLVEEPQNLIKQ | EFKP-VEEP------ | 306.00 |
| 401 | FKPLVEEPQNLIKQN | ---LVEEPQ------ | 78.00 |
| 402 | KPLVEEPQNLIKQNC | --LVEEPQ------- | 67.00 |
| 403 | PLVEEPQNLIKQNCE | -LVEEPQN------- | 96.00 |
| 404 | LVEEPQNLIKQNCEL | LVEEPQ--------- | 89.00 |
| 405 | VEEPQNLIKQNCELF | --------------- | 64.00 |
| 406 | EEPQNLIKQNCELFE | --------------- | 63.00 |
| 407 | EPQNLIKQNCELFEQ | --------------- | 60.00 |
| 408 | PQNLIKQNCELFEQL | --------------- | 66.00 |
| 409 | QNLIKQNCELFEQLG | --------------- | 60.00 |
| 410 | NLIKQNCELFEQLGE | --------------- | 65.00 |
| 411 | LIKQNCELFEQLGEY | --------------- | 71.00 |
| 412 | IKQNCELFEQLGEYK | --------------- | 67.00 |
| 413 | KQNCELFEQLGEYKF | ---------L-EY-F | 85.00 |
| 414 | QNCELFEQLGEYKFQ | ---ELFE-LGEYKFQ | 145.00 |
| 415 | NCELFEQLGEYKFQN | --E-FE-LGEY-FQ- | 139.00 |
| 416 | CELFEQLGEYKFQNA | -ELFE-LGEYKFQ-- | 135.00 |
| 417 | ELFEQLGEYKFQNAL | --FEQLGEY-FQ--- | 189.00 |
| 418 | LFEQLGEYKFQNALL | LFE-LGEY-FQ---- | 165.00 |
| 419 | FEQLGEYKFQNALLV | -E-L-EY-F------ | 99.00 |
| 420 | EQLGEYKFQNALLVR | ----E---------- | 70.00 |
| 421 | QLGEYKFQNALLVRY | ---E----------- | 65.00 |
| 422 | LGEYKFQNALLVRYT | --------------- | 52.00 |
| 423 | GEYKFQNALLVRYTK | --------------- | 42.00 |
| 424 | EYKFQNALLVRYTKK | --------------- | 41.00 |
| Posi-tion | Peptide | Epitope | Native Signal |
| 425 | YKFQNALLVRYTKKV | --------------- | 36.00 |
| 426 | KFQNALLVRYTKKVP | --------------- | 33.00 |
| 427 | FQNALLVRYTKKVPQ | --------------- | 36.00 |
| 428 | QNALLVRYTKKVPQV | --------------- | 38.50 |
| 429 | NALLVRYTKKVPQVS | --------------- | 41.00 |
| 430 | ALLVRYTKKVPQVST | --------------- | 43.00 |
| 431 | LLVRYTKKVPQVSTP | --------------- | 42.50 |
| 432 | LVRYTKKVPQVSTPT | --------------- | 39.00 |
| 433 | VRYTKKVPQVSTPTL | --------------- | 40.00 |
| 434 | RYTKKVPQVSTPTLV | --------------- | 34.50 |
| 435 | YTKKVPQVSTPTLVE | --------------- | 43.00 |
| 436 | TKKVPQVSTPTLVEV | --------------- | 43.00 |
| 437 | KKVPQVSTPTLVEVS | --------------- | 37.00 |
| 438 | KVPQVSTPTLVEVSR | -----------E--- | 51.50 |
| 439 | VPQVSTPTLVEVSRN | --------------- | 62.00 |
| 440 | PQVSTPTLVEVSRNL | --------------- | 63.00 |
| 441 | QVSTPTLVEVSRNLG | --------E----L- | 71.00 |
| 442 | VSTPTLVEVSRNLGK | -------E-S-NL-- | 72.00 |
| 443 | STPTLVEVSRNLGKV | -----VEVSRNLG-- | 77.00 |
| 444 | TPTLVEVSRNLGKVG | -----E-SRNLG--- | 75.00 |
| 445 | PTLVEVSRNLGKVGS | ----E-SRNLG---- | 75.00 |
| 446 | TLVEVSRNLGKVGSK | -----S-NL------ | 54.00 |
| 447 | LVEVSRNLGKVGSKC | --E-S---------- | 49.00 |
| 448 | VEVSRNLGKVGSKCC | -EVS-NL-------- | 45.00 |
| 449 | EVSRNLGKVGSKCCK | --------------- | 32.00 |
| 450 | VSRNLGKVGSKCCKH | --------------- | 27.00 |
| 451 | SRNLGKVGSKCCKHP | --------------- | 25.00 |
| 452 | RNLGKVGSKCCKHPE | --------------- | 26.00 |
| 453 | NLGKVGSKCCKHPEA | --------------- | 29.00 |
| 454 | LGKVGSKCCKHPEAK | --------------- | 26.00 |
| 455 | GKVGSKCCKHPEAKR | --------------- | 24.00 |
| 456 | KVGSKCCKHPEAKRM | ----------E---- | 24.00 |
| 457 | VGSKCCKHPEAKRMP | --------------- | 27.00 |
| 458 | GSKCCKHPEAKRMPC | --------E------ | 27.00 |
| 459 | SKCCKHPEAKRMPCA | --------------- | 27.00 |
| 460 | KCCKHPEAKRMPCAE | --------------- | 26.00 |
| 461 | CCKHPEAKRMPCAED | -----E--------- | 36.00 |
| 462 | CKHPEAKRMPCAEDY | --------------- | 36.00 |
| 463 | KHPEAKRMPCAEDYL | ---E----------- | 40.00 |
| 464 | HPEAKRMPCAEDYLS | --------------- | 43.00 |
| 465 | PEAKRMPCAEDYLSV | -E------------- | 50.00 |
| 466 | EAKRMPCAEDYLSVV | --------------- | 47.00 |
| 467 | AKRMPCAEDYLSVVL | --------------- | 43.00 |
| 468 | KRMPCAEDYLSVVLN | --------------- | 47.00 |
| 469 | RMPCAEDYLSVVLNQ | -----E--LSVV--- | 70.00 |
| 470 | MPCAEDYLSVVLNQL | ----E--L------- | 71.00 |
| 471 | PCAEDYLSVVLNQLC | ---E------L---- | 73.00 |
| 472 | CAEDYLSVVLNQLCV | --E---S-------- | 74.00 |
| 473 | AEDYLSVVLNQLCVL | -E-YLS-V--Q---- | 78.00 |
| 474 | EDYLSVVLNQLCVLH | --------------- | 63.00 |
| 475 | DYLSVVLNQLCVLHE | --------------- | 55.00 |
| 476 | YLSVVLNQLCVLHEK | -------------E- | 53.00 |
| 477 | LSVVLNQLCVLHEKT | ------------E-- | 53.00 |
| 478 | SVVLNQLCVLHEKTP | -----------E--- | 47.00 |
| 479 | VVLNQLCVLHEKTPV | ----------E---- | 49.00 |
| 480 | VLNQLCVLHEKTPVS | --------------- | 47.00 |
| 481 | LNQLCVLHEKTPVSD | --------------- | 50.00 |
| 482 | NQLCVLHEKTPVSDR | -------E------- | 46.00 |
| 483 | QLCVLHEKTPVSDRV | ------E-------- | 51.00 |
| 484 | LCVLHEKTPVSDRVT | -----E--------- | 49.00 |
| Posi-tion | Peptide | Epitope | Native Signal |
| 485 | CVLHEKTPVSDRVTK | --------------- | 41.00 |
| 486 | VLHEKTPVSDRVTKC | --------------- | 44.00 |
| 487 | LHEKTPVSDRVTKCC | --E------------ | 43.00 |
| 488 | HEKTPVSDRVTKCCT | --------------- | 38.50 |
| 489 | EKTPVSDRVTKCCTE | E-------------E | 51.50 |
| 490 | KTPVSDRVTKCCTES | -------------E- | 45.00 |
| 491 | TPVSDRVTKCCTESL | --------------- | 52.00 |
| 492 | PVSDRVTKCCTESLV | -----------E--- | 52.00 |
| 493 | VSDRVTKCCTESLVN | --------------- | 58.00 |
| 494 | SDRVTKCCTESLVNR | ---------E----- | 53.00 |
| 495 | DRVTKCCTESLVNRR | --------E--V--- | 50.00 |
| 496 | RVTKCCTESLVNRRP | --------------- | 42.00 |
| 497 | VTKCCTESLVNRRPC | ------E-------- | 50.50 |
| 498 | TKCCTESLVNRRPCF | -----E--------- | 49.00 |
| 499 | KCCTESLVNRRPCFS | ----E---------- | 50.00 |
| 500 | CCTESLVNRRPCFSA | ---ES---------- | 59.00 |
| 501 | CTESLVNRRPCFSAL | --E------------ | 56.00 |
| 502 | TESLVNRRPCFSALE | --------------- | 59.00 |
| 503 | ESLVNRRPCFSALEV | --------------- | 60.00 |
| 504 | SLVNRRPCFSALEVD | ------------E-- | 62.00 |
| 505 | LVNRRPCFSALEVDE | --------------- | 62.00 |
| 506 | VNRRPCFSALEVDET | --------------- | 61.00 |
| 507 | NRRPCFSALEVDETY | --------LEVDETY | 485.00 |
| 508 | RRPCFSALEVDETYV | -------LEVDETY- | 467.00 |
| 509 | RPCFSALEVDETYVP | ------LEVDETY-- | 550.00 |
| 510 | PCFSALEVDETYVPK | -----LEVDETY--- | 613.00 |
| 511 | CFSALEVDETYVPKE | ----LEVDETY---- | 562.00 |
| 512 | FSALEVDETYVPKEF | ---LEVDETY----- | 496.00 |
| 513 | SALEVDETYVPKEFN | --LE-DETY-P---- | 422.00 |
| 514 | ALEVDETYVPKEFNA | -LEVDETY------- | 500.00 |
| 515 | LEVDETYVPKEFNAE | LEVDETY-------- | 320.00 |
| 516 | EVDETYVPKEFNAET | EVDETYV-------- | 117.00 |
| 517 | VDETYVPKEFNAETF | --------------- | 61.00 |
| 518 | DETYVPKEFNAETFT | --------------- | 62.50 |
| 519 | ETYVPKEFNAETFTF | --------------- | 68.00 |
| 520 | TYVPKEFNAETFTFH | --------------- | 64.00 |
| 521 | YVPKEFNAETFTFHA | --------------- | 63.50 |
| 522 | VPKEFNAETFTFHAD | --------------- | 62.00 |
| 523 | PKEFNAETFTFHADI | --------------- | 56.00 |
| 524 | KEFNAETFTFHADIC | --------------- | 61.00 |
| 525 | EFNAETFTFHADICT | ------F-------- | 66.00 |
| 526 | FNAETFTFHADICTL | ---E----------- | 65.50 |
| 527 | NAETFTFHADICTLS | --E------------ | 64.00 |
| 528 | AETFTFHADICTLSE | --------------- | 56.00 |
| 529 | ETFTFHADICTLSEK | --------------- | 63.00 |
| 530 | TFTFHADICTLSEKE | --------------- | 61.00 |
| 531 | FTFHADICTLSEKER | --------------- | 59.00 |
| 532 | TFHADICTLSEKERQ | --------------- | 64.00 |
| 533 | FHADICTLSEKERQI | F-ADI--LSEKERQI | 114.00 |
| 534 | HADICTLSEKERQIK | ---I-TLSEKERQI- | 137.00 |
| 535 | ADICTLSEKERQIKK | AD---LSEKERQI-- | 128.00 |
| 536 | DICTLSEKERQIKKQ | ----LSEKE-QI--- | 170.00 |
| 537 | ICTLSEKERQIKKQT | ---LSEKE-QI---- | 137.00 |
| 538 | CTLSEKERQIKKQTA | -TLSEKE-QI---TA | 131.00 |
| 539 | TLSEKERQIKKQTAL | -LSEKE-QI--QT-- | 137.00 |
| 540 | LSEKERQIKKQTALV | LSEKE-QI---T--- | 135.00 |
| 541 | SEKERQIKKQTALVE | ---E----------- | 52.00 |
| 542 | EKERQIKKQTALVEL | --------------- | 52.00 |
| 543 | KERQIKKQTALVELV | --------------- | 50.00 |
| 544 | ERQIKKQTALVELVK | --------------- | 53.00 |
| Posi-tion | Peptide | Epitope | Native Signal |
| 545 | RQIKKQTALVELVKH | --------------- | 40.00 |
| 546 | QIKKQTALVELVKHK | --------------- | 37.00 |
| 547 | IKKQTALVELVKHKP | --------------- | 35.50 |
| 548 | KKQTALVELVKHKPK | --------------- | 31.00 |
| 549 | KQTALVELVKHKPKA | --------------- | 33.00 |
| 550 | QTALVELVKHKPKAT | --------------- | 37.00 |
| 551 | TALVELVKHKPKATK | --------------- | 35.00 |
| 552 | ALVELVKHKPKATKE | --------------- | 37.00 |
| 553 | LVELVKHKPKATKEQ | --------------- | 40.00 |
| 554 | VELVKHKPKATKEQL | --------------- | 39.00 |
| 555 | ELVKHKPKATKEQLK | -L------------- | 19.00 |
| 556 | LVKHKPKATKEQLKA | --------------- | 22.00 |
| 557 | VKHKPKATKEQLKAV | --------------- | 16.00 |
| 558 | KHKPKATKEQLKAVM | --------------- | 13.50 |
| 559 | HKPKATKEQLKAVMD | --------------- | 22.50 |
| 560 | KPKATKEQLKAVMDD | ------E--KA--D- | 39.00 |
| 561 | PKATKEQLKAVMDDF | -----E-L-A--DD- | 38.00 |
| 562 | KATKEQLKAVMDDFA | ----E---------- | 38.00 |
| 563 | ATKEQLKAVMDDFAA | -TKEQLKA--DDF-- | 97.00 |
| 564 | TKEQLKAVMDDFAAF | --E-LKA--DDF--- | 71.00 |
| 565 | KEQLKAVMDDFAAFV | -E---A--DD----- | 56.00 |
| 566 | EQLKAVMDDFAAFVE | E--------F--F-- | 57.00 |
| 567 | QLKAVMDDFAAFVEK | -------------E- | 41.00 |
| 568 | LKAVMDDFAAFVEKC | -----D------E-- | 42.00 |
|  |  |  |  |
| Posi-tion | Peptide | Epitope | Native Signal |
| 569 | KAVMDDFAAFVEKCC | -----------E--- | 33.00 |
| 570 | AVMDDFAAFVEKCCK | ----------E---- | 37.00 |
| 571 | VMDDFAAFVEKCCKA | ---D-----E----- | 36.00 |
| 572 | MDDFAAFVEKCCKAD | --------E------ | 34.00 |
| 573 | DDFAAFVEKCCKADD | -------E------- | 43.00 |
| 574 | DFAAFVEKCCKADDK | D-----E------D- | 31.00 |
| 575 | FAAFVEKCCKADDKE | -----E--------E | 32.00 |
| 576 | AAFVEKCCKADDKET | ----E---------- | 32.00 |
| 577 | AFVEKCCKADDKETC | ---E-----DD-ET- | 41.00 |
| 578 | FVEKCCKADDKETCF | --E-----DD-E--- | 42.00 |
| 579 | VEKCCKADDKETCFA | -E-C---DD-ETC-- | 60.00 |
| 580 | EKCCKADDKETCFAE | -----ADD-ETCF-E | 108.00 |
| 581 | KCCKADDKETCFAEE | -----DD-ETC--EE | 90.00 |
| 582 | CCKADDKETCFAEEG | C---DD-ETC--EE- | 114.00 |
| 583 | CKADDKETCFAEEGK | ---DD-ET---EE-- | 78.50 |
| 584 | KADDKETCFAEEGKK | ---D-E----EE--- | 38.00 |
| 585 | ADDKETCFAEEGKKL | --D------E----- | 48.00 |
| 586 | DDKETCFAEEGKKLV | ---E----E------ | 41.50 |
| 587 | DKETCFAEEGKKLVA | D------EE------ | 38.00 |
| 588 | KETCFAEEGKKLVAA | -E----E-------- | 27.00 |
| 589 | ETCFAEEGKKLVAAS | E-----E-------- | 36.00 |
| 590 | TCFAEEGKKLVAASQ | ----EE--------- | 33.00 |
| 591 | CFAEEGKKLVAASQA | ---E----------- | 31.00 |
| 592 | FAEEGKKLVAASQAA | --E------------ | 31.00 |
| 593 | AEEGKKLVAASQAAL | -----------E--- | 29.00 |

**S1 Table: Target-specific positions of individual peptides**. The table shows output of all overlapping peptides mapping the HSA protein sequence. Positions identified as being important for binding (identified by the Dunnett’s test of complete single-amino acid substitutions at the p<0.0001 level) are highlighted whereas dashes indicate positions not involved in binding. The median signal of copies of the corresponding native peptide is shown.
