# Supplementary material for: ArrayPitope: Automated Analysis of Amino Acid Substitutions for Peptide Microarray-Based Antibody Epitope Mapping
Source: PLoS One. 2017 Jan 17;12(1):e0168453. doi: 10.1371/journal.pone.0168453 (PMC5240915; doi:10.1371/journal.pone.0168453)
Supplement: S2 Table — Table showing 12 HSA epitope regions identified by the algorithm. The dataset was limited to only include alanine substitutions of native peptides of HSA. Dashes mark gaps of residues with no selectivity. The regions were identified from the logo plots (not shown), and defined as having a minimum of 4 residues and a maximum gap-length of 2 residues. (DOC) [file pone.0168453.s003.doc]

| **Range** | **Region** |
| --- | --- |
| 35-41 | F-DLGEE |
| 66-76 | LVNEVTEF--T |
| 80-96 | DES-E--DKSL-TLF-D |
| 101-106 | V-T-RE |
| 151-159 | F-DNEETFL |
| 202-212 | LLP-LDEL--E |
| 230-235 | FGE--F |
| 245-263 | Q-FP--EF-E-S-LV--LT |
| 320-337 | DEMP-DL--L--DFVE-K |
| 398-410 | FDEFKP-VEEP-N |
| 419-428 | FE-LGE--FQ |
| 515-521 | LEVDETY |
| 540-547 | LSEKE--I |

**S2 Table. HSA epitopes found through substitution with alanine only.** Table showing 12 HSA epitope regions identified by the algorithm. The dataset was limited to only include alanine substitutions of native peptides of HSA. Dashes mark gaps of residues with no selectivity. The regions were identified from the logo plots (not shown), and defined as having a minimum of 4 residues and a maximum gap-length of 2 residues.
